# Towards InAs/InGaAs/GaAs Quantum Dot Solar Cells Directly Grown on Si Substrate

**DOI:** 10.3390/ma8074544

**Published:** 2015-07-22

**Authors:** Bilel Azeza, Mohamed Helmi Hadj Alouane, Bouraoui Ilahi, Gilles Patriarche, Larbi Sfaxi, Afif Fouzri, Hassen Maaref, Ridha M’ghaieth

**Affiliations:** 1 Laboratoire Micro-Optoélectroniques et Nanostructures, Faculté des Sciences de Monastir, Université de Monastir, Monatir 5019, Tunisie; E-Mails: helmi.alouane@lpn.cnrs.fr (M.H.H.A.); larbi.sfaxi@fsm.rnu.tn (L.S.); hassen.maaref@fsm.rnu.tn (H.M.); ridha.mghaieth@fsm.rnu.tn (R.M.); 2 Turaif Sciences College, Northern Borders University, P.O. 833, Turaif 91411, Kingdom of Saudi Arabia; 3 Laboratoire de Photonique et de Nanostructures (LPN), UPR20-CNRS, Route de Nozay, Marcoussis 91460, France; E-Mail: gilles.patriarche@lpn.cnrs.fr; 4 King Saud University, Department of Physics and Astronomy, College of Sciences, P.O. 2455, Riyadh 11451, Kingdom of Saudi Arabia; E-Mail: bilahi@ksu.edu.sa; 5 Laboratoire de Physico-Chimie des Matériaux, Faculté des Sciences de Monastir, Université de Monastir, Monastir 5019, Tunisie; E-Mail: afif.fouzri@fsm.rnu.tn

**Keywords:** III-V materials for solar cells, Si substrate, molecular beam epitaxy, solar cell

## Abstract

This paper reports on an initial assessment of the direct growth of In(Ga)As/GaAs quantum dots (QDs) solar cells on nanostructured surface Si substrate by molecular beam epitaxy (MBE). The effect of inserting 40 InAs/InGaAs/GaAs QDs layers in the intrinsic region of the heterojunction pin-GaAs/n^+^-Si was evaluated using photocurrent spectroscopy in comparison with pin-GaAs/n^+^-Si and pin-GaAs/GaAs without QDs. The results reveal the clear contribution of the QDs layers to the improvement of the spectral response up to 1200 nm. The novel structure has been studied by X ray diffraction (XRD), photoluminescence spectroscopy (PL) and transmission electron microscopy (TEM). These results provide considerable insights into low cost III-V material-based solar cells.

## 1. Introduction

Recent attempts have been made to increase solar cell efficiency by exploiting the below band gap photon energies. This approach is often termed as impurity band solar cells or intermediate band solar cells (IBSC) [1,2,3,4]. This type of structure demonstrates an enhancement of the spectral response towards lower photon energies with promising possibilities to improve the solar cell’s efficiency up to 63% according to theoretical expectation [5,6,7,8]. However, the main problem encountered for III-V element-based solar cells mainly lies in the development of large surface photovoltaic structures due to their high cost, making the employment of low cost substrates highly desirable. Accordingly, the epitaxial growth of the GaAs layer on Si substrate has attracted considerable attention owing to their large area availability, low cost and high mechanical strength [9,10,11,12,13,14]. Promising InAs/GaAs QD-based optoelectronic devices, directly grown on Si substrate, have already been reported [15,16,17,18,19,20]. In the meantime, with the exception of the employment of the bonding technique [21], the feasibility of the direct growth of InAs/GaAs QDs solar cells on Si substrate has not yet been explored.

In this context, this paper reports, for the first time, on the effect of inserting InAs/InGaAs/GaAs multiple QDs layers within the pin-GaAs structure directly deposited by MBE on nanostructured Si substrate for solar cell applications. The results may open up new perspectives on the development of low cost, high efficiency III-V-based solar cells on Si substrate.

## 2. Results and Discussion

### 2.1. Growth Process

InAs/GaAs QDs with In_0.13_Ga_0.87_As strain reducing layer were incorporated within the intrinsic region of a pin-GaAs/n^+^-Si using Stranski-Kranstanow growth mode by molecular beam epitaxy. The use of InGaAs as a strain reducing layer is believed both to reduce the compressive stress acting on the InAs QDs by the GaAs matrix, and to reduce indium out-diffusion from the InAs QDs [22,23,24]. The choice of relatively low indium composition is expected to avoid excessive In-Ga phase separation that alters the optical and structural properties of the InAs QDs and the surrounding material [25]. The number of absorbed photons is proportional to the number of effective QDs in the solar cell active region. For the InAs/GaAs system, the QDs’ aerial density in a single layer is around 10^10^ dots/cm^2^ on GaAs substrate [26]. Such a value is too small to account for the improvement of the spectral response. Increasing the effective number of QDs is possible by vertical stacking of QD layers. However, the total number of vertically stacked layers is limited by the onset required for the relaxation of the accumulated strain by generation of stacking faults and dislocations. In the present study, we have employed 40 QD layers ensuring a compromise between the increase of the effective number of QDs and the overall sample’s structural properties. The pin diode structure has been directly fabricated on nanostructured n^+^-Si substrate.

The preparation of the nanostructured n+-type silicon’s substrate surface has been performed at room temperature by the formation and subsequent dissolution of a porous layer. The electrolyte used to fabricate a 5 µm-thick porous silicon layer consists of a mixture of hydrofluoric acid HF ([HF] = 36%) and ethanol (HF:C2H5OH) in a volumetric proportion of 1:1. The porous layer was formed by anodizing the Si substrate in this electrolyte under a current density of 3 mA·cm^−2^. The sample was then etched in NaOH solution to break up the porous silicon layer and produce the structuration of the surface. Indeed, after the chemical dissolution of silicon skeleton, the rugged surface will be exposed to beam epitaxy. Additional details concerning the process as well as the morphological properties of the nanostructured Si surface and its impact on the quality of GaAs material grown on such Si surface can be found elsewhere [27].

After surface preparation, a cleaning and out gassing process of the silicon substrate was performed under vacuum condition in an introductory chamber with a rest pressure of 10^−9^ Torr at high temperature (760 °C), to remove the native oxide and other volatile compounds prior to the GaAs deposition. The growth began by depositing 0.25 µm n+ doped GaAs layer at 530 °C followed by 1 µm of n doped GaAs layer at 580 °C. Forty layers of 0.7 nm nominal thickness InAs QDs capped first by 5 nm In_0.13_Ga_0.87_As and then 7 nm GaAs spacer layer were subsequently deposited at 500 °C. Finally, 0.5 µm p doped GaAs was grown at 580 °C followed by 0.1 µm of p^+^ doped GaAs layer at 530 °C. The growth rate was: 0.24 Ås^−1^ for the InAs, 2.22 Ås^−1^ for the InGaAs and 1.98 Ås^−1^ for GaAs.

**Figure 1:**
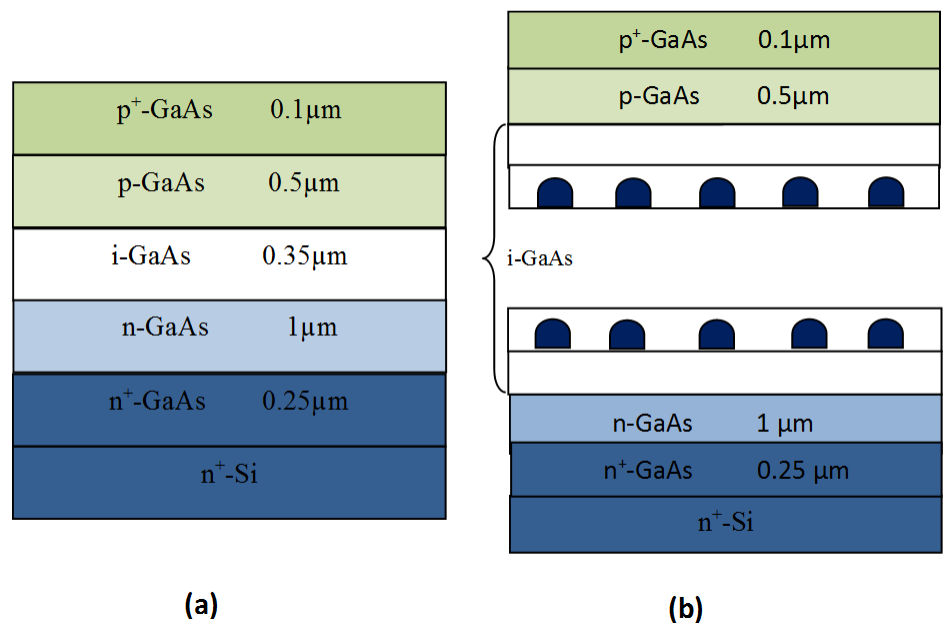
Schematic presentation of the investigated samples. (**a**) pin-GaAs/Si; (**b**) pin-GaAs/Si containing 40 QD layers.

Two reference pin-GaAs diodes without QDs have also been fabricated under the same conditions either on nanostructured Si substrate and GaAs substrate. A schematic presentation of the pin-GaAs diode on Si substrate with and without QDs is given in Figure 1.

**Figure 2:**
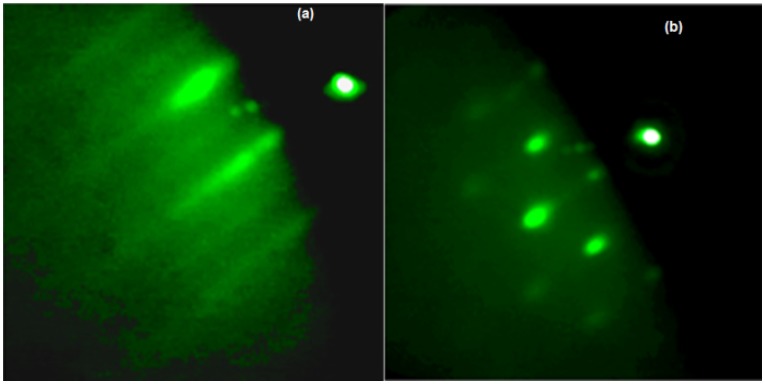
RHEED diffraction pattern. (**a**) During the growth of GaAs; (**b**) after the deposition of the InAs QDs.

During the growth process, the surface morphology was *in-situ* monitored by reflection high-energy electron diffraction (RHEED). As shown by Figure 2, the RHEED pattern changed from streaky (Figure 2a) during the GaAs deposition, which is characteristic of 2D growth mode to a spotty pattern (Figure 2b) after the deposition of InAs material. The observed changes in the diffraction pattern represent the transition from 2D to 3D growth mode, testifying the QDs’ formation.

### 2.2. Material Characterization

**Figure 3:**
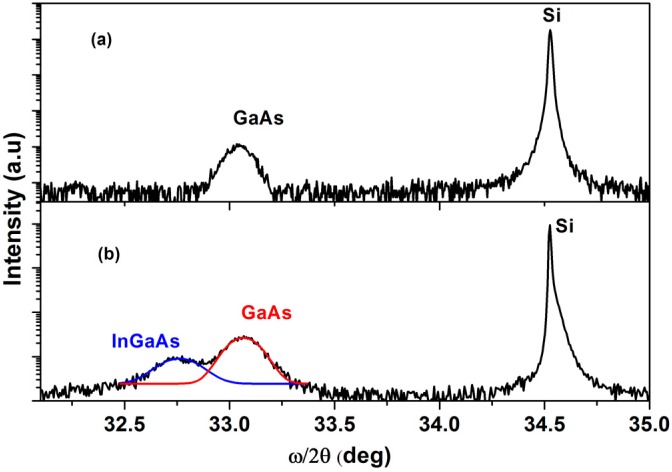
The ω/2θ of reflection peak of: (**a**) pin-GaAs/ Si; (**b**) pin-GaAs/Si with InAs/InGaAs multilayer QDs.

Figure 3 illustrates the ω/2θ, reflection peaks of pin-GaAs/n^+^-Si with and without InAs/InGaAs multistaked QDs.

**Figure 4:**
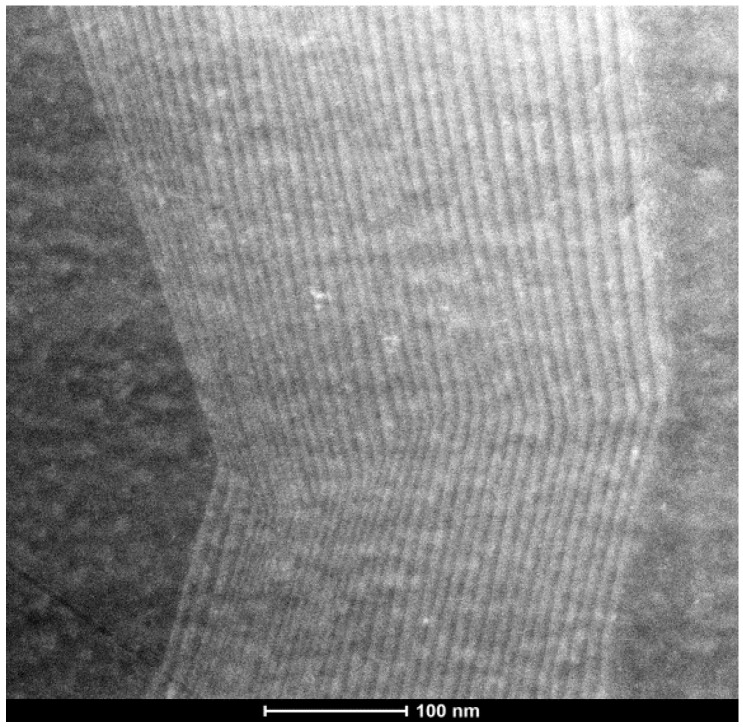
Cross section TEM image of the InAs/InGaAs multilayer QDs.

The spectra from the pin-GaAs (a) and pin-GaAs with QDs (b) grown on Si substrate reveal the presence of two peaks centered on θ = 34.52° and θ = 33.06° and attributed respectively to the silicon substrate and to the GaAs layer. Additionally, a third peak appear at θ = 32.75° in the XRD spectra of the structure containing the QDs. This peak can be attributed to the InAs/InGaAs multilayer with a nominal indium composition x_m_ equal to the average of indium compositions in all layers (x_m_ = 13.27% estimated by HRXRD). For the InAs/GaAs multistaked QDs grown on GaAs substrate, the HRXRD spectra show the appearance of other peaks appointed satellite peaks, due to the periodicity introduced by the bilayers repetition and the angular period of this peak is related to the thickness of the bilayer [28,29]. In our case, the absence of satellite peaks could be explained by the existence of defects produced in the interfaces layers. Indeed, as shown by the Figure 4, the cross section transmission electron microscopy image unambiguously shows that the GaAs buffer layer was not sufficiently smooth. The surface roughness greatly influenced the multiple layer QDs, resulting in distorted layers. Consequently, the grown InAs/InGaAs QDs display a non-uniform thickness which in turn provokes plastic strain relaxation via defects and threading dislocations.

**Figure 5:**
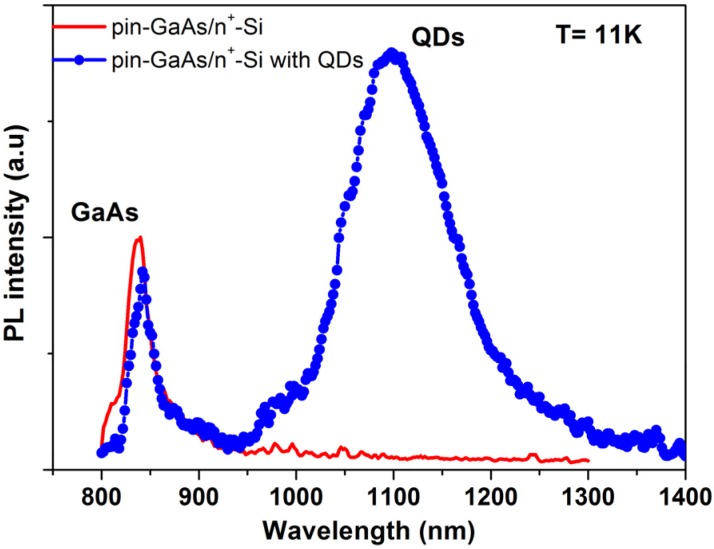
PL spectra recorded at 11 k from the pin-GaAs/Si structure (red line) and from pin-GaAs/Si with InAs/InGaAs multilayer QDs (blue line).

Additional details can be given by PL characterization. Figure 5 shows the 11 K PL spectra of the pin-GaAs/n^+^-Si structures with and without QDs. A peak centered at 842 nm appear in both structures and are attributed to the GaAs’ emission. The red shift of GaAs emission peak is a consequence of the lattice mismatch between GaAs and Si, the polar/nonpolar character and of the strong tensile stress, since the thermal expansion coefficient of GaAs is about twice that of the silicon value. The low intensity of these peaks is directly linked to the subsistence of non-radiative recombination channels due to the defects in the structure.

For the structure containing multiple layer QDs, the PL measurement reveals a broad band centered at 1100 nm. This band is likely to arise from the luminescence of the InAs QDs. Although this result confirms the formation of InAs/GaAs QDs, the broadening of the PL band with relatively weak intensity confirms that the QDs structural properties are altered.

**Figure 6:**
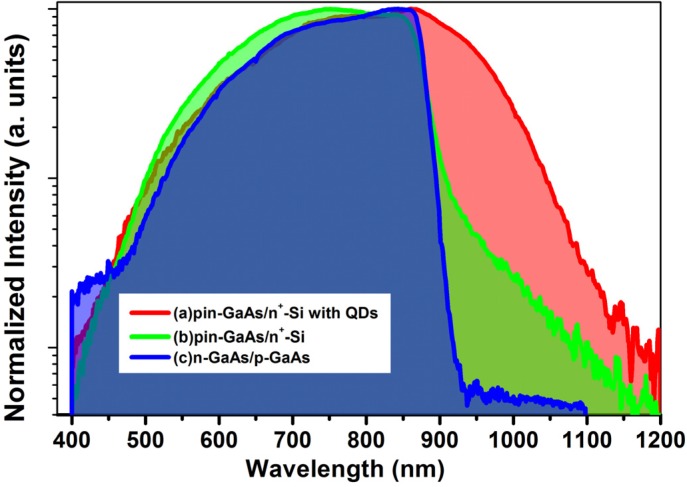
Spectral response of (**a**) pin-GaAs/ n^+^-Si with QDs (red); (**b**) pin-GaAs/n^+^-Si without QDs (green); (**c**) reference pin-GsAs on GaAs substrate (blue).

To further assess the impact of introducing the InAs QDs within the pin-GaAs/Si we have performed the spectral response measurements from samples with and without QDs. The results are shown by Figure 6. The photo-response obtained from pin-GaAs/n^+^-Si without QDs for the high energy photons (beyond the GaAs band gap) produces the same range of photo-response obtained by the reference cell grown on GaAs substrate. This assures that the photocarriers collected by the structure are mainly created by the pin-GaAs prepared on the Si substrate. However, for lower energy photons, the spectral response of the reference cell drops abruptly at 868 nm corresponding to the band gap energy of GaAs (1.42 eV). In the meantime, the photo-response from pin-GaAs/n^+^-Si recovers a proportion of the below GaAs band gap photons to an extent of up to 1200 nm. The observed enhancement is due to photocarriers generated by silicon substrate.

A more pronounced improvement in the photo-response at long wavelengths is observed for the structure containing QDs. This improvement is due to the absorption of photons below the band gap energy of GaAs by InAs QDs.

Although the structural properties of the multiple QDs appear rather to be degraded principally as a consequence of the initial surface roughness, the optical and electric properties of pin-GaAs/n+-Si with InAs QDs show that the InAs QDs have been formed and successively contribute to the electron–hole pair creations in the below band gap energy range which increased the photocarrier collections [30,31]. This initial assessment provides evidence of the potential of our proposed yielding structures for the fabrication of future novel, low cost, high performance solar cells.

At this time, no contact grid coatings were applied and the electrical contact was basically made with indium-zinc alloys pads on the front surface. *In-situ* and *ex-situ* optimization of the solar cell fabrication is in progress, a necessary step to obtain significant values from the active solar cell parameters.

## 3. Experimental Section

The HRXRD experiments were performed with D8 DISCOVER Bruker Axs Diffractometer (BRUKER, Karlsruhe, Germany) with CuKα1 radiation (λ CuKα = 1.5406Å) for ω/2θ values in the range of 32°–35° to investigate the structural properties of GaAs layer grown on nanostructured Si substrate.

The PL measurements have been done at 11 K, and the samples mounted in a closed cycle He cryostat, were excited with the 514.5 nm line of an Ar^+^ laser ( Spectra-Physics, Santa Clara, CA, USA) while the spectra were collected using a thermoelectrically cooled InGaAs photodetector (Oriel, Stratford, CT, USA) using a conventional lock-in technique.

The cross section transmission electron microscopy image was performed using a TEM/STEM Cs-corrected JEOL 2200 FS (JEOL, Peabody, MA, USA) operated at 200 kV.

The spectral response measurements aim to evaluate the electrical current photogenerated in our samples. The spectral response is measured using a 100 W tungsten halogen lamp (Newport, Santa Clara, CA, USA), CVI CM110 1/8 m monochromator (Spectral Product, Cvijovica Dolina, CA, USA) and a lock-in amplifier connected to a chopper (at 172 Hz) placed at the outlet of the source monochromator.

## 4. Conclusions

InAs/GaAs QD-based pin GaAs solar cells directly grown on silicon substrate have been demonstrated for the first time by using the nanostructured surface as a buffer layer. This initial assessment shows the formation of InAs nanostructure, with an emission wavelength of around 1100 nm. The insertion of multiple layer QDs within a pin GaAs grown on Si substrate has been found to improve the spectral response to 1200 nm, despite imperfect structural properties. These results hold great promise for future demonstration of high efficiency IBSCs on Si substrate via heteroepitaxy using nanostructured surfaces.
